# Pulsatile Proptosis due to Intraorbital Meningocele

**DOI:** 10.3389/fneur.2017.00290

**Published:** 2017-06-19

**Authors:** Anouke van Rumund, Aad Verrips, Wim I. M. Verhagen

**Affiliations:** ^1^Department of Neurology, Radboud University Medical Center, Nijmegen, Netherlands; ^2^Department of Neurology, Canisius-Wilhelmina Hospital, Nijmegen, Netherlands

**Keywords:** proptosis, orbital roof fracture, meningocele, pulsations, MRI imaging

## Abstract

We present a case of a 79-year-old man with a non-symptomatic pulsatile proptosis of the left eye. Magnetic resonance imaging revealed a meningocele into the left orbit due to an osseous defect in the orbital roof.

## Introduction

Basal encephalo- or meningoceles are rare, approximately 1.5% of all cases, and intraorbital encephalo- or meningoceles are even more rare. The most common causes are trauma, congenital skull malformations, and tumors (1). We present a patient with pulsatile exophthalmos due to an intraorbital meningocele.

## Case Report

**Figure 1:**
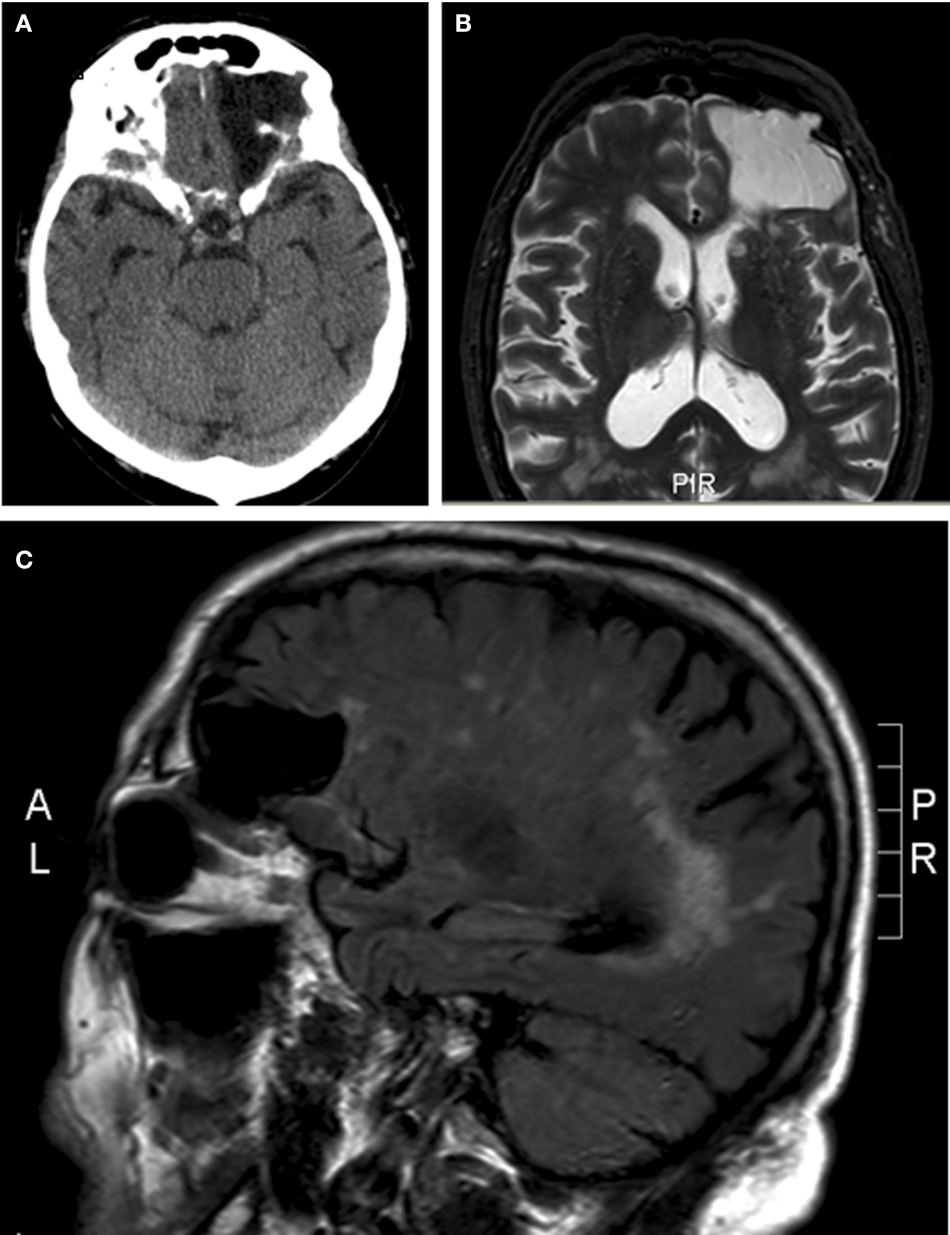
Meningocele into the left orbit due to a bony defect in the orbital roof. **(A)** Axial computed tomography. **(B)** Axial magnetic resonance imaging short T1 inversion recovery sequence. **(C)** Sagittal magnetic resonance imaging, fluid-attenuated inversion recovery sequence.

A 79-year-old man presented with a transient ischemic attack of the posterior circulation. He had no complaints at that time. On neurological examination, he had a non-symptomatic, pulse-synchronous pulsatile proptosis of the left eye (see Video S1 in Supplementary Material). According to the patient, this was present since childhood or even birth. There was no complaint of oscillopsia. He denied a history of birth trauma or head injury. He had no history of congenital anomalies, bone dysplasia, or neurofibromatosis. The neurological examination was otherwise normal and no bruit was heard. There was 4 mm proptosis of the left eye. Visual acuity without correction was for OD 1.0 and for OS 0.4. Intraocular pressure was 9 mmHg in OD and 10 mmHg in OS. Direct and indirect pupillary responses were normal. OD showed pseudophakia. OS had cataract. There was no conjunctival venous congestion nor venous congestion of the posterior poles of the eyes. Arterial abnormalities were absent in the posterior pole of the eye. He had full range eye movements without double vision. Examination of the eyes was further unremarkable. Computed tomography and MR imaging (see Figure 1) revealed a meningocele into the left orbit due to a bony defect in the orbital roof (42 mm × 37 mm). He was not bothered by the proptosis and declined surgical correction of the orbital roof.

## Discussion

The differential diagnosis of pulsatile proptosis includes orbital roof fractures, encephalo- or meningoceles, neurosurgical procedures (1–3), neurofibromatosis type 1, and vascular malformations such as carotid-cavernous fistula and arteriovenous malformations (4, 5). Even in orbital roof fractures, pulsatile proptosis is rare (2, 3). Our patient had only a meningocele into the left orbital due to a bony defect of the orbital roof, but no history of any of the other options mentioned above. Pulsation of the brain blood vessels passed on to the CSF explains the synchrony of the eyeball pulsation to the arterial pulse. We hypothesize that he has had head injury in early childhood leading to an orbital roof fracture and posttraumatic meningocele or a congenital skull base defect. In the literature, surgery is recommended especially for late onset traumatic encephaloceles with improvement of the preoperative ocular symptoms in all patients (2, 3). However, our patient declined surgery due to the fact that he had no symptoms and only signs.

## Ethics Statement

This is a case report. The patient approved publication.

## Author Contributions

AR wrote the clinical information of the patient. All authors participated in the description of the images, the introduction, discussion, and abstract.

## Conflict of Interest Statement

The authors declare that the research was conducted in the absence of any commercial or financial relationships that could be construed as a potential conflict of interest.

## Supplementary Material

Video S1 Pulsatile proptosis of the left eye.
